# Implementation of Machine Learning Pipelines for Clinical Practice: Development and Validation Study

**DOI:** 10.2196/37833

**Published:** 2022-12-16

**Authors:** Lara J Kanbar, Benjamin Wissel, Yizhao Ni, Nathan Pajor, Tracy Glauser, John Pestian, Judith W Dexheimer

**Affiliations:** 1 Division of Pulmonary Medicine Cincinnati Children's Hospital Medical Center Cincinnati, OH United States; 2 Division of Biomedical Informatics Cincinnati Children's Hospital Medical Center Cincinnati, OH United States; 3 Department of Pediatrics University of Cincinnati College of Medicine Cincinnati, OH United States; 4 Division of Neurology Cincinnati Children's Hospital Medical Center Cincinnati, OH United States; 5 Division of Emergency Medicine Cincinnati Children's Hospital Medical Center Cincinnati, OH United States

**Keywords:** electronic health record, natural language processing, epilepsy, clinical decision support, machine learning, emergency medicine, artificial intelligence

## Abstract

**Background:**

Artificial intelligence (AI) technologies, such as machine learning and natural language processing, have the potential to provide new insights into complex health data. Although powerful, these algorithms rarely move from experimental studies to direct clinical care implementation.

**Objective:**

We aimed to describe the key components for successful development and integration of two AI technology–based research pipelines for clinical practice.

**Methods:**

We summarized the approach, results, and key learnings from the implementation of the following two systems implemented at a large, tertiary care children’s hospital: (1) epilepsy surgical candidate identification (or epilepsy ID) in an ambulatory neurology clinic; and (2) an automated clinical trial eligibility screener (ACTES) for the real-time identification of patients for research studies in a pediatric emergency department.

**Results:**

The epilepsy ID system performed as well as board-certified neurologists in identifying surgical candidates (with a sensitivity of 71% and positive predictive value of 77%). The ACTES system decreased coordinator screening time by 12.9%. The success of each project was largely dependent upon the collaboration between machine learning experts, research and operational information technology professionals, longitudinal support from clinical providers, and institutional leadership.

**Conclusions:**

These projects showcase novel interactions between machine learning recommendations and providers during clinical care. Our deployment provides seamless, real-time integration of AI technology to provide decision support and improve patient care.

## Introduction

With the rampant growth in health data, artificial intelligence (AI) technologies, such as machine learning and natural language processing (NLP), provide a powerful means to extract meaningful associations from big data sets [1]. Applications of machine learning are far-reaching and have included patient identification, computer vision, speech recognition, web searches, and phenotype discovery [2-9].

The electronic health record (EHR) captures data relating to clinical encounters, but as much as 30%-50% of these data are available only in free text [10]. As such, one particularly valuable means to understand health care data involves NLP. NLP is a technique of incorporating free-text analysis and statistical methods into computerized algorithms to derive linguistic features (eg, physicians’ diagnosis) from human language input [11]. Clinical care and research can benefit from using this unstructured text information [12,13]. NLP has been used for surveillance, adverse event detection [14-18], medication identification [19], and extraction of data from radiology reports [20-22]. NLP has also successfully been applied to evaluate clinical notes and provide recommendations as part of clinical decision support (CDS) tools [23].

These CDS tools can change user behavior; however, to ensure successful implementation, user involvement in CDS design is critical [24-30]. CDS tools using AI and NLP technologies remain less implementable directly into real-time clinical care with long-term success [31-34]. The reason integration of these AI pipelines within a clinical health system is challenging is that it requires coordination with the following: (1) key stakeholders and expected end users of the CDS tools; (2) biomedical informatics professionals who design the AI; (3) research information technology (IT) professionals who design the CDS tools with stakeholders in mind; and (4) operational IT professionals who are responsible for maintenance, uptime, and EHR integration [35].

In this work, we report the main modifications implemented to improve the development and real-time integration of two AI technology–based pipelines using NLP in a tertiary pediatric health care institution. These modifications contributed to the successful deployment and ongoing utilization of these pipelines.

## Methods

### Objective

The objective of our case studies was to create functional AI technology–based CDS tools effective in research settings and integrate them into clinical workflow without sacrificing care quality, speed of clinical care delivery, and labor requirement.

### Setting and Participants

Cincinnati Children’s Hospital Medical Center is a large tertiary care center with more than 1.2 million patient encounters annually. It has a large epilepsy clinic (over 6,400 patients and 12,000 epilepsy visits per year) and a high volume of epilepsy surgery cases (50 per year). The division of pediatric emergency medicine oversees 5 urgent cares and 2 emergency departments (EDs) with an annual census of 170,000 visits. The ED employs 8 full-time clinical research coordinators (CRCs) who enroll patients in research studies during clinical visits.

### Case 1: Automated Epilepsy Interventions

#### Background

The first case study aimed to facilitate early surgical intervention in patients with intractable epilepsy, as it has been shown to improve cognitive outcomes, mental health, and quality of life [36], as well as increase quality-adjusted life years [37] in a relatively safe procedure for the patient [38]. National guidelines state that patients who continue to have debilitating seizures after 2 or more adequate trials of antiepileptic medications should be considered for a presurgical evaluation referral [39]. From the time of first seizure, on average, patients receive surgery after having epilepsy for 7 years in pediatrics and 20 years in adults [40,41]. Only 0.5%-1.5% of patients received surgery within 2 years of fulfilling clinical criteria for surgical candidacy [42]. Indeed, improving the use of surgery has proven to be difficult [42] because this highly specialized but critical clinical knowledge is not ubiquitously available in clinical care.

#### Approach

**Figure 1:**
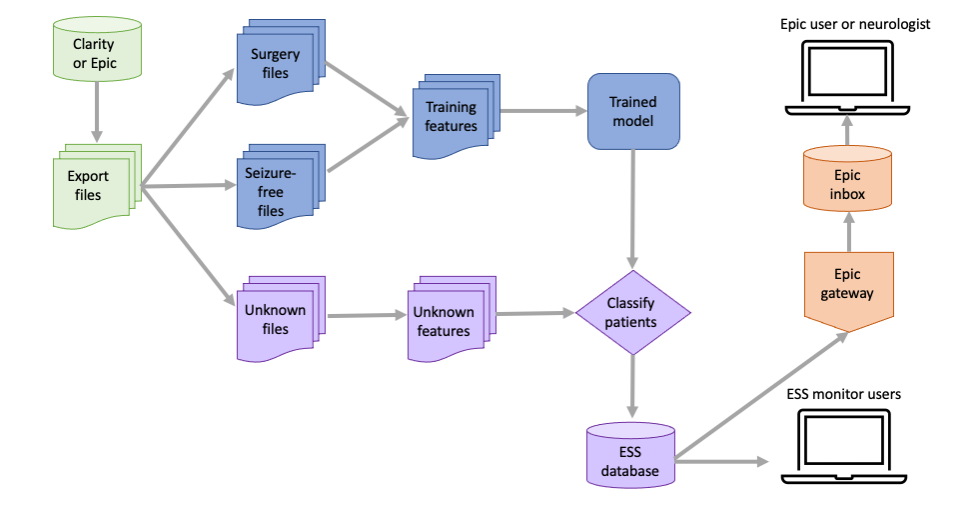
Epilepsy surgical pipeline architecture. From left to right: a series of Oracle PL/SQL queries extract epilepsy patient data and export them in CSV format to bare meta installation servers. The data are divided into the following 3 groups: patients with surgery, seizure-free patients, and patients with unknown outcome. The feature extraction module (ie, ‘training features’) analyzes the free-text notes and exports machine-readable feature vectors in SVM light format. Surgery and seizure-free patient features are sent to the classifier training module to train the support vector machine model. Unknown patient features are fed into the final trained classifier, which outputs a surgery candidacy score for each patient. All patients with unknown outcome and their scores are then loaded into the Epilepsy Surgery Software (ESS) database. The highest scoring patients are sent to an Epic web service that generates the in-basket message alerts. All patients and their notes can be viewed and searched in the ESS web application. This entire process is run on a weekly basis, to continually incorporate new electronic health record data into the algorithm training.

A corpus of notes from patients with a diagnosis of epilepsy who were seizure free or had a history of resective epilepsy surgery was used to devise NLP features. The NLP generated surgical candidacy scores for each patient, with higher scores indicating a higher likelihood of surgical candidacy and lower scores indicating a higher likelihood of seizure freedom. Next, naïve Bayes, support vector machine, and random forest models were developed using retrospective data as described in previous work [43]. Figure 1 describes the system pipeline from input data to the output recommendation.

To ensure the recommendations from the NLP system would be accepted into practice, we validated the algorithm’s classifications by comparing them head-to-head against manual labels from epileptologists [2]. Prior to implementation into clinical care, we prospectively evaluated the system for 1 year to test the accuracy in a clinical setting [44].

### Case 2: Automated Clinical Trial Eligibility Screener (ACTES)

#### Background

The second case study aimed to identify participants who may meet eligibility criteria for clinical trial recruitment in the ED. In current practice, CRCs and physicians at the site of the hospitals do trial eligibility screening manually [45]. For patients presenting during clinical visits, screening would ideally take place early enough in the visit such that eligible candidates could be approached for enrollment without prolonging their length of stay. However, given the large volume of data documented in EHRs, it is labor-intensive for the staff to screen relevant information, particularly within the time frame of a single visit. As such, automatically screening and identifying eligible patients for a trial based on EHR information promises great benefits for clinical research.

#### Approach

To facilitate participant identification, we developed a machine learning NLP-based system—ACTES [23,46]—which analyzed structured data and unstructured narrative notes automatically to determine patients’ suitability for clinical trial enrollment. For development, we evaluated historical trial-patient enrollment decisions in a pediatric ED and extracted EHR data including clinical notes that were commonly reviewed by CRCs. We then customized the machine learning and NLP algorithms based on the trial-patient data. The ACTES was integrated into the institutional workflow to support real-time patient screening in our recent work [44]; details of system development have been previously reported [46].

### Implementation Strategy

We hypothesized that successful implementation of the AI solutions relied on 5 key steps, as follows:

1. Integration of industry standard software pertinent to the implementation site. Specifically, the systems needed to be adapted to use industry standard software libraries.
2. Automation of the process to access the EHR data. The systems need to be linked to the EHR to extract the input data without manual intervention.
3. Encouragement of user feedback to inform the final design of the AI solution.
4. Integration of the AI solutions into typical clinical workflow.
5. Performance evaluations and regular maintenance to continue to evaluate the utility of the AI solution.

After building the AI technology, we implemented the AI solutions using these 5 strategies to facilitate successful deployment of the tools.

## Results

After creation and validation of the algorithms in a research setting, we implemented these 2 AI solutions as NLP pipelines. Both pipelines follow a step-by-step process that extracts data from the EHR, processes it, and provides a recommendation in the form of automated alerts that could be sent from the research systems to the EHR (Epic Systems) in real time. To do this, the research systems had to be modified to integrate into clinical workflow, as described in this section.

### Industry Standard Software

After reviewing over 20 different libraries for managing NLP pipelines, it was decided that the Java NLP library LingPipe [47] would be used for feature extraction and preprocessing, and the LIBSVM Python implementation from scikit-learn [48] would be used for the classifier [49]. The NLP component in ACTES was built upon the clinical Text Analysis and Knowledge Extraction System [50], and the machine learning component was coded in Java (Oracle Corporation).

### Automation of EHR Data Access

For the epilepsy intervention AI, Oracle PL/SQL queries from the EHR relational database were used to extract patient data. For ACTES, RESTful and SOAP web services were developed to extract EHR data, such as demographics, medication orders, and clinical notes in real time, which were stored in an Oracle SQL database. An interactive web-based dashboard was developed to visualize the recommendations and receive feedback from CRCs.

### User Feedback Informed the Final Design

AI solutions were designed and integrated with feedback from end users. The epilepsy and ACTES corpora were created by manual annotation of patient notes by providers. Throughout the algorithm design and implementation process, providers were included in the build and ultimate integration. First, the biomedical informatics team shadowed providers for workflow observation. Second, the biomedical informatics team attended clinical meetings that included faculty, staff, and clinical research coordinators for a minimum of 10 hours to get feedback and ensure the design was appropriate. Third, mock-up designs were shared at a minimum of 3 meetings to discuss the process of using and interacting with the AI solution in the form of a CDS tool. In cases where the CDS tool could provide an alert, the providers were consulted on their preferred alert method (eg, email or text message alerts). In both AI technologies, the providers were able to directly interact with the machine learning recommendations as follows:

- For epilepsy surgical intervention, these results are displayed in clinical care to suggest surgical consults, and the subsequent actions resulting from the recommendations are fed back into the application to improve performance.
- For ACTES, the clinical research coordinators’ entry of eligibility is used to help train and improve the classifier. Additionally, ACTES was assessed and improved for usability and satisfaction by providers and was found to be easy to use and learn.

### Integration Into Clinical Workflow

Both AI technologies were integrated into clinical workflow to support clinical practice. For patients with intractable epilepsy and an upcoming visit, surgical eligibility is evaluated in advance. For patients who are classified as potential surgical candidates, EHR in-basket messages are sent to the provider they are scheduled to see via web services.

We integrated the ACTES into the CRCs’ workflow to support real-time patient screening [51]. The system ran continuously on a secured, The Health Insurance Portability and Accountability Act (HIPAA)–compliant server to extract structured and unstructured EHR data for current ED patients. For each clinical trial, the ranked list of patients recommended by the system, along with their demographics and clinical information, were displayed on the dashboard available to the CRCs. The information was refreshed at 10-minute increments to accommodate real-time updates. Given the recommended patients as potential participants for a clinical trial, the CRCs performed additional EHR screening to confirm the candidates’ eligibility. When an eligible candidate was identified, the CRCs approached him or her for enrollment as per standard clinical workflow.

### Performance Evaluation

The epilepsy AI technology went live on April 12, 2016, as part of the EHR release cycle and runs weekly. On Sundays, the system trains on notes from patients who have been seizure free for 1 year or previously underwent resective epilepsy surgery. This trained classifier evaluates all other ‘unknown’ patients with epilepsy who have had at least one seizure within the last year but have not had a presurgical evaluation. Thus, the tables of training and test patients are updated weekly. The system performs as well as board-certified neurologists in identifying surgical candidates (with a sensitivity of 71% and positive predictive value of 77%) and improves with additional training, identifying surgical candidates faster than neurologists [2]. As part of the ongoing algorithmic development, the number of patients with a history of surgery included in the training set increased from 102 patients on April 10, 2016, to 195 patients on October 6, 2019.

The ACTES patient identification system went live on October 1, 2017. ACTES was prospectively evaluated using a time-and-motion study, quantitative assessments of enrollment, and postevaluation usability surveys collected from the CRCs [52]. During the time-and-motion study, an observer monitored the activities a CRC was engaged in at 30-second increments for 2 hours. The time spent per activity was compared to that prior to the use of ACTES. This study was repeated monthly for 4 months, and it was distributed among CRCs and shifts. After the implementation of ACTES, the CRCs spent 12.9% (*P*<.001) less time on electronic screening [52]. The quantitative assessments of enrollment evaluated the number of patients screened, the number of patients approached, and the number of patients enrolled. The use of ACTES significantly improved the number of screened patients for the majority of trials and improved the number of approached patients and enrolled patients, with statistical significance in 2 of 7 trials [52]. Finally, results from the System Usability Survey and additional open-ended questions were analyzed on a monthly basis to improve ACTES [52].

### Maintenance

The epilepsy system was operational more than 90% of the time through the first 150 weeks. Throughout this time, issues were addressed by the biomedical informatics research and production IT staff. There were 10 changes made to the NLP system and 6 errors executing the pipeline of scripts. Issues extracting patient notes from the EHR were the largest reason for delays in running the NLP system, which occurred 12 out of 150 (8%) weeks.

Miscellaneous adjustments were made to the ACTES tool during the pilot phase (2017-2018) to accommodate CRC needs. ACTES was also updated 3 times because of significant updates on the institutional EHR system and its web services for real-time data extraction. Updates on the institutional EHR system and the research IT environment caused multiple system breakdowns during the evaluation period that interrupted less than 2 out of 52 (4%) weeks of operation.

## Discussion

### Principal Results

This work highlighted the major modifications for the integration and deployment of CDS tools from the research setting to clinical practice. We successfully added AI-based technology to the following 2 distinct clinical workflows at our institution: an automated epilepsy surgical intervention tool and an automated clinical trial eligibility screener (ACTES) system. Throughout the process, we determined that successful integration of these tools into clinical care requires adaptation to industry standards, automation of data access, logical integration into clinical workflow, and continual user feedback.

This work has several important strengths. We implemented novel, automated machine learning tools to provide decision support in a tangible fashion at our institution. These tools were well received and streamlined clinical care in the identification of qualified patients for surgery or clinical trials. Our experience with the deployment of these tools agreed with the suggestions made by Kawamoto et al [53] for successful implementation of CDS tools. Our CDS tools were implemented in real time to provide support at a natural point in the clinical workflow, so as not to disrupt or extend the timeline of care. As with their findings, our CDS tools use automatically available EHR data, where possible, to ensure clinical scalability and effective usability. In our case, we added an extra layer of testing whereby we implemented our CDS tools in a localized clinical setting in parallel to clinical care to test accuracy prior to full deployment, which allowed for continued fine-tuning of the CDS tool before it became part of clinical workflow.

### Evaluation of Bias

We evaluated both tools for potential bias to ensure that the CDS recommendations were not influenced by racial disparities. The AI technology behind epilepsy surgical candidacy recommendation was evaluated for bias in terms of patient demographics, socioeconomic characteristics, and language [54]. Patient race, gender, and primary language did not bias the AI’s surgical candidacy scores (*P*>.35 for all).

### Considerations and Limitations

Several concerns should be considered in the implementation of a research tool into real-time clinical settings. As with most record keeping systems, the EHR systems require regular upgrades and bug fixes. This necessitates ongoing IT support to keep the pipeline operational. EHR algorithm extractions and pipeline characteristics should be placed into the EHR upgrade queue to ensure their evaluation with each upgrade cycle. To account for this, resources for both operational and research IT should be set aside to ensure a consistent system when integrated with clinical practice.

The successful deployment and continued use of these systems also required close collaboration with the stakeholders embedded in the respective clinical system. This collaboration was crucial in allowing seamless integration of the research output into daily clinical practice. Without input from the effective end users, it would be difficult to fully understand the current process, needs, as well as limitations related to workflow and data to allow for optimization of the prediction.

### Conclusions

The formulation, development, and real-time implementation of two AI solutions in a clinical setting required the development of a CDS tool and pipeline using public, industry-standard programs and existing EHR web interfaces prior to integration. In our work, we found that a CDS tool’s success was largely dependent upon the collaboration between machine learning experts, research collaborators, and operational IT professionals. Furthermore, longitudinal support from clinical providers and institutional leadership is necessary for continued maintenance of the deployed CDS tool with careful consideration for its long-term use.

## Abbreviations

ACTES: automated clinical trial eligibility screener
AI: artificial intelligence
CDS: clinical decision support
CRC: clinical research coordinator
ED: emergency department
EHR: electronic health record
IT: information technology
NLP: natural language processing

## References

[1] Rajkomar A, Dean J, Kohane I (2019). Machine learning in medicine. N Engl J Med.

[2] Cohen KB, Glass B, Greiner HM, Holland-Bouley K, Standridge S, Arya R, Faist R, Morita D, Mangano F, Connolly B, Glauser T, Pestian J (2016). Methodological issues in predicting pediatric epilepsy surgery candidates through natural language processing and machine learning. Biomed Inform Insights.

[3] Matykiewicz P, Cohen K, Holland KD, Glauser TA, Standridge SM, Verspoor KM, Pestian J (2013). Earlier identification of epilepsy surgery candidates using natural language processing.

[4] Zhang X, Kim J, Patzer RE, Pitts SR, Patzer A, Schrager JD (2017). Prediction of emergency department hospital admission based on natural language processing and neural networks. Methods Inf Med.

[5] Zeng Z, Shi H, Wu Y, Hong Z (2015). Survey of natural language processing techniques in bioinformatics. Comput Math Methods Med.

[6] Milea D, Najjar RP, Jiang Z, Ting D, Vasseneix C, Xu X, Aghsaei Fard M, Fonseca P, Vanikieti K, Lagrèze WA, La Morgia C, Cheung CY, Hamann S, Chiquet C, Sanda N, Yang H, Mejico LJ, Rougier M, Kho R, Tran TH, Singhal S, Gohier P, Clermont-Vignal C, Cheng C, Jonas JB, Yu-Wai-Man P, Fraser CL, Chen JJ, Ambika S, Miller NR, Liu Y, Newman NJ, Wong TY, Biousse V (2020). Artificial intelligence to detect papilledema from ocular fundus photographs. N Engl J Med.

[7] Gulshan V, Peng L, Coram M, Stumpe MC, Wu D, Narayanaswamy A, Venugopalan S, Widner K, Madams T, Cuadros J, Kim R, Raman R, Nelson PC, Mega JL, Webster DR (2016). Development and validation of a deep learning algorithm for detection of diabetic retinopathy in retinal fundus photographs. JAMA.

[8] Tomašev N, Glorot X, Rae JW, Zielinski M, Askham H, Saraiva A, Mottram A, Meyer C, Ravuri S, Protsyuk I, Connell A, Hughes CO, Karthikesalingam A, Cornebise J, Montgomery H, Rees G, Laing C, Baker CR, Peterson K, Reeves R, Hassabis D, King D, Suleyman M, Back T, Nielson C, Ledsam JR, Mohamed S (2019). A clinically applicable approach to continuous prediction of future acute kidney injury. Nature.

[9] Esteva A, Kuprel B, Novoa RA, Ko J, Swetter SM, Blau HM, Thrun S (2017). Dermatologist-level classification of skin cancer with deep neural networks. Nature.

[10] Hicks J (2003). The potential of claims data to support the measurement of health care quality. RAND Corporation.

[11] Hirschberg J, Manning CD (2015). Advances in natural language processing. Science.

[12] Melton GB, Hripcsak G (2005). Automated detection of adverse events using natural language processing of discharge summaries. J Am Med Inform Assoc.

[13] Murff HJ, FitzHenry F, Matheny ME, Gentry N, Kotter KL, Crimin K, Dittus RS, Rosen AK, Elkin PL, Brown SH, Speroff T (2011). Automated identification of postoperative complications within an electronic medical record using natural language processing. JAMA.

[14] Bates DW, Evans RS, Murff H, Stetson PD, Pizziferri L, Hripcsak G (2003). Detecting adverse events using information technology. J Am Med Inform Assoc.

[15] Petratos GN, Kim Y, Evans RS, Williams SD, Gardner RM (2017). Comparing the effectiveness of computerized adverse drug event monitoring systems to enhance clinical decision support for hospitalized patients. Appl Clin Inform.

[16] Tinoco A, Evans RS, Staes CJ, Lloyd JF, Rothschild JM, Haug PJ (2011). Comparison of computerized surveillance and manual chart review for adverse events. J Am Med Inform Assoc.

[17] Conway M, Dowling JN, Chapman WW (2013). Using chief complaints for syndromic surveillance: a review of chief complaint based classifiers in North America. J Biomed Inform.

[18] Ye Y, Tsui F, Wagner M, Espino JU, Li Q (2014). Influenza detection from emergency department reports using natural language processing and Bayesian network classifiers. J Am Med Inform Assoc.

[19] Savova GK, Olson JE, Murphy SP, Cafourek VL, Couch FJ, Goetz MP, Ingle JN, Suman VJ, Chute CG, Weinshilboum RM (2012). Automated discovery of drug treatment patterns for endocrine therapy of breast cancer within an electronic medical record. J Am Med Inform Assoc.

[20] Dublin S, Baldwin E, Walker RL, Christensen LM, Haug PJ, Jackson ML, Nelson JC, Ferraro J, Carrell D, Chapman WW (2013). Natural Language Processing to identify pneumonia from radiology reports. Pharmacoepidemiol Drug Saf.

[21] Elkin PL, Froehling D, Wahner-Roedler D, Trusko B, Welsh G, Ma H, Asatryan AX, Tokars JI, Rosenbloom ST, Brown SH (2008). NLP-based identification of pneumonia cases from free-text radiological reports. AMIA Annu Symp Proc.

[22] Friedman C, Alderson PO, Austin JHM, Cimino JJ, Johnson SB (1994). A general natural-language text processor for clinical radiology. J Am Med Inform Assoc.

[23] Deleger L, Brodzinski H, Zhai H, Li Q, Lingren T, Kirkendall ES, Alessandrini E, Solti I (2013). Developing and evaluating an automated appendicitis risk stratification algorithm for pediatric patients in the emergency department. J Am Med Inform Assoc.

[24] Branch-Elliman W, Strymish J, Kudesia V, Rosen AK, Gupta K (2015). Natural language processing for real-time catheter-associated urinary tract infection surveillance: results of a pilot implementation trial. Infect Control Hosp Epidemiol.

[25] Tso GJ, Tu SW, Oshiro C, Martins S, Ashcraft M, Yuen KW, Wang D, Robinson A, Heidenreich PA, Goldstein MK (2016). Automating guidelines for clinical decision support: knowledge engineering and implementation. AMIA Annu Symp Proc.

[26] Klein ME, Parvez MM, Shin J (2017). Clinical implementation of pharmacogenomics for personalized precision medicine: barriers and solutions. J Pharm Sci.

[27] Kilsdonk E, Peute LW, Jaspers MWM (2017). Factors influencing implementation success of guideline-based clinical decision support systems: a systematic review and gaps analysis. Int J Med Inform.

[28] Castillo RS, Kelemen A (2013). Considerations for a successful clinical decision support system. Comput Inform Nurs.

[29] Garg AX, Adhikari NKJ, McDonald H, Rosas-Arellano MP, Devereaux PJ, Beyene J, Sam J, Haynes RB (2005). Effects of computerized clinical decision support systems on practitioner performance and patient outcomes: a systematic review. JAMA.

[30] Wright A, Ash JS, Aaron S, Ai A, Hickman TT, Wiesen JF, Galanter W, McCoy AB, Schreiber R, Longhurst CA, Sittig DF (2018). Best practices for preventing malfunctions in rule-based clinical decision support alerts and reminders: results of a Delphi study. Int J Med Inform.

[31] Abràmoff MD, Lavin PT, Birch M, Shah N, Folk JC (2018). Pivotal trial of an autonomous AI-based diagnostic system for detection of diabetic retinopathy in primary care offices. NPJ Digit Med.

[32] Hollon TC, Pandian B, Adapa AR, Urias E, Save AV, Khalsa SSS, Eichberg DG, D'Amico RS, Farooq ZU, Lewis S, Petridis PD, Marie T, Shah AH, Garton HJL, Maher CO, Heth JA, McKean EL, Sullivan SE, Hervey-Jumper SL, Patil PG, Thompson BG, Sagher O, McKhann GM, Komotar RJ, Ivan ME, Snuderl M, Otten ML, Johnson TD, Sisti MB, Bruce JN, Muraszko KM, Trautman J, Freudiger CW, Canoll P, Lee H, Camelo-Piragua S, Orringer DA (2020). Near real-time intraoperative brain tumor diagnosis using stimulated Raman histology and deep neural networks. Nat Med.

[33] Titano JJ, Badgeley M, Schefflein J, Pain M, Su A, Cai M, Swinburne N, Zech J, Kim J, Bederson J, Mocco J, Drayer B, Lehar J, Cho S, Costa A, Oermann EK (2018). Automated deep-neural-network surveillance of cranial images for acute neurologic events. Nat Med.

[34] Wang P, Liu X, Berzin Tm, Glissen Brown Jr, Liu P, Zhou C, Lei L, Li L, Guo Z, Lei S, Xiong F, Wang H, Song Y, Pan Y, Zhou G (2020). Effect of a deep-learning computer-aided detection system on adenoma detection during colonoscopy (CADe-DB trial): a double-blind randomised study. Lancet Gastroenterol Hepatol.

[35] Trinkley KE, Kahn MG, Bennett TD, Glasgow RE, Haugen H, Kao DP, Kroehl ME, Lin C, Malone DC, Matlock DD (2020). Integrating the practical robust implementation and sustainability model with best practices in clinical decision support design: implementation science approach. J Med Internet Res.

[36] Engel J, McDermott MP, Wiebe S, Langfitt JT, Stern JM, Dewar S, Sperling MR, Gardiner I, Erba G, Fried I, Jacobs M, Vinters HV, Mintzer S, Kieburtz Karl, Early Randomized Surgical Epilepsy Trial (ERSET) Study Group (2012). Early surgical therapy for drug-resistant temporal lobe epilepsy: a randomized trial. JAMA.

[37] Choi H, Sell RL, Lenert L, Muennig P, Goodman RR, Gilliam FG, Wong JB (2008). Epilepsy surgery for pharmacoresistant temporal lobe epilepsy: a decision analysis. JAMA.

[38] Engel J, Wiebe S, French J, Sperling M, Williamson P, Spencer D, Gumnit R, Zahn C, Westbrook E, Enos B (2003). Practice parameter: temporal lobe and localized neocortical resections for epilepsy. Epilepsia.

[39] Cross JH, Jayakar P, Nordli D, Delalande O, Duchowny M, Wieser HG, Guerrini R, Mathern GW, International League against Epilepsy‚ Subcommission for Paediatric Epilepsy Surgery, Commissions of NeurosurgeryPaediatrics (2006). Proposed criteria for referral and evaluation of children for epilepsy surgery: recommendations of the Subcommission for Pediatric Epilepsy Surgery. Epilepsia.

[40] Choi H, Carlino R, Heiman G, Hauser WA, Gilliam FG (2009). Evaluation of duration of epilepsy prior to temporal lobe epilepsy surgery during the past two decades. Epilepsy Res.

[41] Kwan P, Schachter SC, Brodie MJ (2011). Drug-resistant epilepsy. N Engl J Med.

[42] Englot DJ, Ouyang D, Garcia PA, Barbaro NM, Chang EF (2012). Epilepsy surgery trends in the United States, 1990-2008. Neurology.

[43] Tsochantaridis I, Hofmann T, Thorsten J, Altun Y (2004). Support vector machine learning for interdependent and structured output spaces. Proceedings of the twenty-first international conference on Machine learning.

[44] Wissel BD, Greiner HM, Glauser TA, Holland-Bouley Katherine D, Mangano FT, Santel D, Faist R, Zhang N, Pestian JP, Szczesniak RD, Dexheimer JW (2020). Prospective validation of a machine learning model that uses provider notes to identify candidates for resective epilepsy surgery. Epilepsia.

[45] Embi PJ, Payne PRO (2009). Clinical research informatics: challenges, opportunities and definition for an emerging domain. JAMIA.

[46] Ni Y, Kennebeck S, Dexheimer JW, McAneney CM, Tang H, Lingren T, Li Q, Zhai H, Solti I (2015). Automated clinical trial eligibility prescreening: increasing the efficiency of patient identification for clinical trials in the emergency department. J Am Med Inform Assoc.

[47] Baldwin B, Dayanidhi K (2014). Natural language processing with Java and LingPipe Cookbook.

[48] Pedregosa F, Varoquaux G, Gramfort A, Michel V, Thirion B, Grisel O, Blondel M, Louppe G, Prettenhofer P, Weiss R, Weiss RJ, Vanderplas J, Passos A, Cournapeau D, Brucher M, Perrot M, Duchesnay E (2011). Scikit-learn: machine learning in Python. J Mach Learn Res.

[49] Joachims Th (1998). Text categorization with support vector machines: learning with many relevant features.

[50] Savova GK, Masanz JJ, Ogren PV, Zheng J, Sohn S, Kipper-Schuler KC, Chute CG (2010). Mayo clinical Text Analysis and Knowledge Extraction System (cTAKES): architecture, component evaluation and applications. J Am Med Inform Assoc.

[51] Dexheimer JW, Tang H, Kachelmeyer A, Hounchell M, Kennebeck S, Solti I, Ni Y (2019). A time-and-motion study of clinical trial eligibility screening in a pediatric emergency department. Pediatr Emerg Care.

[52] Ni Y, Bermudez M, Kennebeck S, Liddy-Hicks S, Dexheimer J (2019). A real-time automated patient screening system for clinical trials eligibility in an emergency department: design and evaluation. JMIR Med Inform.

[53] Kawamoto K, Houlihan CA, Balas EA, Lobach DF (2005). Improving clinical practice using clinical decision support systems: a systematic review of trials to identify features critical to success. BMJ.

[54] Wissel BD, Greiner HM, Glauser TA, Mangano FT, Santel D, Pestian JP, Szczesniak RD, Dexheimer JW (2019). Investigation of bias in an epilepsy machine learning algorithm trained on physician notes. Epilepsia.

